# Survival From Ninety-Five Percent Total Body Surface Area Burn: A Case Report and Literature Review

**DOI:** 10.7759/cureus.21903

**Published:** 2022-02-04

**Authors:** Maisa A AlAlwan, Hussain A Almomin, Shashank D Shringarpure, Nazia U Habiba, Abdulraheim H Albess, Ayyappan Thangavel, Nabil N Youssef, Faisal A Al Jabr, Aqeel H Alrashid, Rayan A Buhalim, Fahad K Almulhim

**Affiliations:** 1 Plastic Surgery, King Fahad Hospital, Hofuf, Al-Ahsa, SAU; 2 College of Medicine, King Faisal University, Al-Ahsa, SAU

**Keywords:** outcome, management, survival, mortality, burns

## Abstract

Burns can be devastating and result in unwanted consequences with prolonged length of hospital stay. The mortality rate increases as the total body surface area increases, so proper management of patients with extensive degrees of burns is crucial for their survival. We present the hospital course, management, and survival of a patient after he sustained a 95% total body surface area, second-degree burn from a gas flame. Furthermore, we present from the literature different cases of patients with large total body surface area burns and survived after being managed in burns specialty centers. Although large total body surface area burns can result in significant morbidity and mortality, early management and intervention by an expert surgical team can result in positive outcomes.

## Introduction

Burns are injuries to the skin and organic tissue that can result in a major threat if not treated properly [1]. Burns are categorized into several types: scald injuries, flame injuries, contact burns, chemical exposure, electrical burns, and radiation injury [1,2]. The mortality in burns is estimated to be 180,000 deaths per year worldwide [3]. Non-fatal burn injuries result in major morbidities, such as infection, systemic inflammatory response syndrome, sepsis, organ failure, acute kidney injury, acute respiratory distress syndrome (ARDS), and lung involvement from inhalation injury that requires mechanical ventilation (MV) [1]. The major predictors of morbidity and mortality of burns include age as well as total body surface area (TBSA) of burns [1,4]. In Saudi Arabia, a systemic review was conducted and found that 80-100% of burns are estimated to be less than 40% TBSA [5]. Furthermore, it is estimated that burns of TBSA more than 40% are associated with major morbidity and mortality [6]. A dramatic decrease in the mortality and morbidity of burns is attributed to the advancement in early resuscitation, advancement in critical care, and early excision and grafting [7]. There is a controversy in terms of how to deal with a patient with large TBSA burns, and there is no consensus about a standardized management plan [8]. There are no previous studies that have presented such a case of TBSA and survived in Saudi Arabia. In this report, we aim to describe our initial burn evaluation, resuscitation phase, and overall management that was done for a patient who sustained a 95% TBSA burn.

## Case presentation

A 24-year-old Somali male patient presented to the emergency room (ER) following a gas flame burn. Per ER report, the patient came to the ER with a companion assistant, and he was conscious, oriented, and spontaneously breathing with no signs of respiratory distress. As for the initial evaluation, the patient’s temperature was 39 °Celsius, tachycardiac with a 140 pulse rate, respiration was 26 breaths per minute, and hypotensive with a blood pressure of 71/50 mmHg. The entire body has been burned except for a very small part of the foot, with TBSA that was estimated to be approximately 95%, which is second-degree superficial. The patient was started with oxygen by oxygen mask, Ringer's lactate 1L, and the initial medical protocol of burn management was initiated including pethidine 100 mg intravenously (IV), metoclopramide 10 mg IV, hydrocortisone 100 mg IV, plasma 500 ml IV, midazolam 3 mg IV, fentanyl 100 mg IV, and followed by mechanical ventilation. Fluid replacement was monitored using a central venous catheter by the help of an anesthesiologist and adjusted according to the urine output to maintain it approximately of more than 0.5 to 1 ml/hr. Upon admission, baseline laboratory investigations were taken, as shown in Table 1.

**Table 1:** Laboratory parameters upon admission and after resuscitation ER: Emergency Room; pCO_2_: Partial pressure of carbon dioxide; pO_2_: Partial pressure of oxygen; FO2Hb: Fraction of Oxyhemoglobin; K^+^: Potassium; Na^+^: Sodium; ctO2: Concentration of Total Oxygen; p50: Oxygen tension when hemoglobin is 50% saturated with oxygen; Base(Ecf): Base excess of extracellular fluid; HCO3- (P.st): Standardized plasma bicarbonate concentration; T: Temperature modified; c: Calculated value(s); e: Estimated value(s)

	Upon ER admission	1 hour post ER admission and resuscitation
Blood Gas Values		
pH	7.181	7.231
pCO_2_	47.8 mmHg	45.8 mmHg
pO_2_	38.1 mmHg	54.1 mmHg
Oximetry Values		
_ct_Hb	23.6 g/dL	19.3 g/dL
sO_2_	61.1%	83.1%
FO_2_Hb_e_	60.6%	82.4%
Electrolyte Values		
cK^+^	4.0 mmol/L	4.6 mmol/L
cNa^+^	136 mmol/L	136 mmol/L
Metabolite Values		
cGlucose	12.5 mmol/L	7.0 mmol/L
cLactate	5.2 mmol/L	9.2 mmol/L
Temperature Corrected Values		
pH (T)	7.181	7.231
pCO_2_ (T)	47.8 mmHg	45.8 mmHg
pO_2_ (T)	38.1 mmHg	54.1 mmHg
Oxygen Status		
ctO_2e_	20.0 Vol%	22.2 Vol%
p50_e_	32.37 mmHg	30.93 mmHg
Acid Base Status		
_c_Base(Ecf)_c_	-9.7 mmol/L	-7.7 mmol/L
_c_HCO_3_^-^ (P.st)_c_	14.6 mmol/L	17.1 mmol/L

Following ER management

Escharotomy was immediately performed for all four limbs, with the application of wound dressing. Then the patient was kept nothing per oral (NPO) overnight. Blood transfusions and four units of warm fresh frozen plasma (FFP) were ordered as well. The patient was moved to the Intensive Care Unit (ICU), in which he was on MV as well as on inotropic support. The patient had the following active issues: 95% TBSA burns, intravascular hypovolemia with shock, mixed acidosis, hypothermia, and evolving multiorgan disease (MOD).

Upon examination in the ICU

The patient’s vital signs were: 86 pulse rate, 91/52 mmHg for the blood pressure, mean arterial pressure (MAP) 51 mmHg, oxygen saturation was 86%. He was on MV, clinically hypothermic with cold extremities, equal bilateral air entry, with escharotomy on all four limbs, and the abdomen was difficult to assess. The urine output was approximately 70 ml/hour, however, the amount of urine output was decreasing gradually within a period of a few hours and it became more concentrated. Laboratory results one day post-admission are shown in Table 2.

**Table 2:** Laboratory results after one day of admission Na^+^: Sodium; K^+^: Potassium; pCO_2_: Partial pressure of carbon dioxide; HCO3-: Bicarbonate

Test	Result	Reference Range
Prothrombin Time	35.2 seconds	11-13.5 seconds
Partial Thromboplastin Time	43.3 seconds	60-70 seconds
International Normalized Ratio (INR)	2.61	0.8-1.1
White Blood Cells	17x10^3^/L	4,000-11,000/μL
Hemoglobin	23.7 g/dL	13.5-17.5 g/dL
Platelets	172x10^3^/L	150,000-450,000/μL
Urea	7.9 mmol/L	1.8-7.1 mmol/L
Creatinine	159 μmol/L	65.4-119.3 μmol/L
Na^+^	135 mEq/L	135-145 mEq/L
K^+^	6.1 mmol/L	3.6-5.2 mmol/L
Calcium	2 mmol/L	2.2-2.6 mmol/L
Alanine aminotransferases	52 U/L	7-56 U/L
Aspartate aminotransferases	46 U/L	10-40 U/L
Total Bilirubin	9.5 mg/dL	0.1-1.2 mg/dL
Serum Albumin	20.6 g/L	34-54 g/L
pH	7.168	7.35-7.45
pCO_2_	55.5 mmHg	35-45 mmHg
HCO_3_^-^	15.2 mmol/L	23-30 mmol/L
Serum Lactate	4.7 mmol/L	Less than 2.3 mmol/L

The patient then was kept NPO, on fentanyl IV infusion 1000 mcg, warmed normal saline that was then changed to 300 ml/hour 5% dextrose solution. Furthermore, inotropes were titrated to keep the MAP more than or equal to 65 mmHg, sodium bicarbonate 8.4% 100 ml IV, ceftriaxone 1g IV for 12 hours. The nephrology department was consulted, and 10 units of FFP and six units of platelets concentrate were ordered.

Three days after admission, the patient developed disseminated intravascular coagulation (DIC), with the international normalized ratio (INR) being 2.00. The patient was treated with four units of warmed FFP. Blood and urine cultures grew gram-negative bacilli 47 days after admission. Sputum, wound, blood, and urine cultures of other days showed no growth. 

Subsequent operations were performed on days 39, 58, and 73 after admission. The first operation was emergent tracheostomy after prolonged intubation, the second operation was left lower eyelid post-burn contraction release, and the last operation was for left lower eyelid ectropion. 

**Figure 1:**
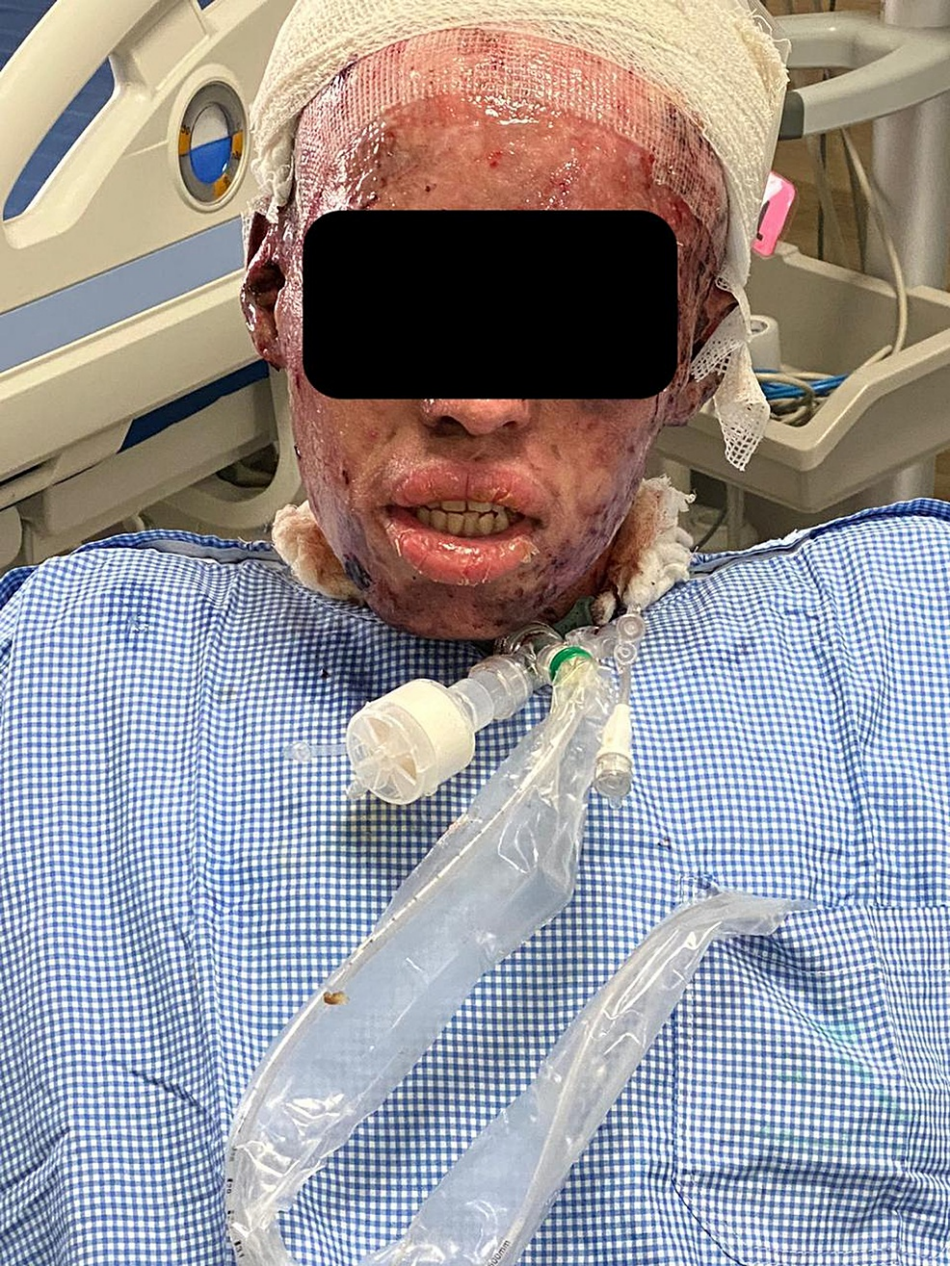
55 days from admission.

**Figure 2:**
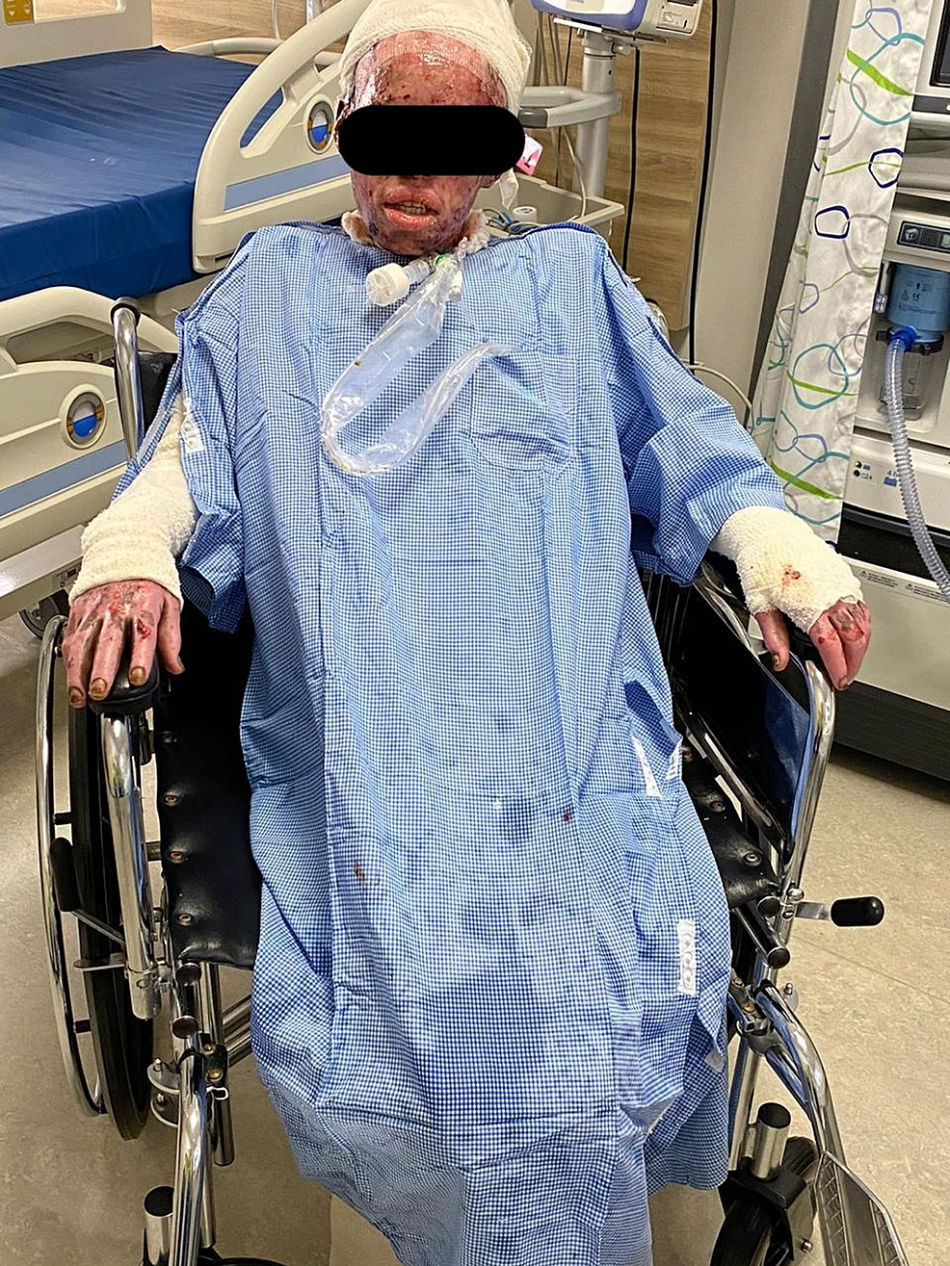
55 days from admission.

The total length of hospital stay was 86 days, with the entire laboratory results during the entire hospital stay mentioned in Table 3. The patient was treated by a physiotherapist and occupational therapist during the hospital stay. He was discharged home after showing continued improvement and lives with his sister to continue improving. Figure 1 and Figure 2 show the patient after 55 days from admission.

**Table 3:** Laboratory results during the entire length of hospital stay > means that test was done several times in the same day as sequential results. WBCs: White Blood Cells; RBC: Red Blood Cells; PT: Prothrombin Time; PTT: Partial Thromboplastin Time; SEC: Second; INR: International Normalized Ratio

Lab/Day	Day1	Day2	Day3	Day4	Day5	Day6	Day7	Day14	Day21	Day28	Day35	Day49	Day 63	Day 86
WBCs [10^9 L]	26.3>20.11> 17.61>10.89 >2.81	2.84	3.32	3.44	1.47	1.88	2.73	6.59> 11.11>3.12	3.38	2.94	5.12	4.12	4.41	4.91
RBC [10^12]	6.77>7.77>7.71>5.87> 4.69	4.96>4.79	4.13	3.56>3.42	3.31	3.25	2.98	2.48>2.78	2.67	2.57	3.59	3.39	3.24	3.8
Hemoglobin [g/dL]	20.6>23.6>23.7>17.8>14.1	14.9>12.5	12.3	10.8>10.3	9.9	9.1	8.9	7.3>8.1	7.1	6.7	9.6	9.6	9.5	11.8
Hematocrit [%]	57.5>65.3>66.5>50.2>39.8	42.5>40.5	35.5	30.9>30.2	29.8	29.6	27.4	22.8>25.5	26.3	21.2	30.2	29.1	29.4	35.5
Platelets [x10^9]	218>191>172>85>50	46>49>45	56	56>71	66>91	94>121>150	173	229>281	353	248	380	198	299	254
Aspartate aminotransferase [U/L]	105>49>46>63>67	84>80	46	35	39>37	31>27	77	98>601>737>587	67>57	35	36	214>56	20	36.1
Alanine transaminase [U/L]	59>52	49	48	35>44	40>29	30>25>47	47	47>133>189>183	47	29	28	104	21	19.1
Direct Bilirubin [umol/L]	0.5>0.1>0>3	3.8>4	3.3	6.2>6	5.5	9>52.9	79.8>64.8>103.2	11.9	18.2	13.9	10.4	9.2	-	2.7
Total Bilirubin [umol/L]	28.5>11.9>9.5>6.2>15.8	18.5>21.4	19.9	24.5>21.6	18.7>27.5	25.5>27.5>70.3	97.9>81.2	23.6>81.4	48.5>48	25.2	24	24.3	10.8	7.2
Urea [mmol/L]	5.2>6.7>7.9>10.5>8.3	5.4>4.8	4.4	5>5.2	4.6>5.2	5.4>5.6	4.7	12.3>10.5	9.4>9.3	7.1	6.8	2.4	5.3	4.2
Creatinine [umol/L]	119.8>136.1>159.3>187.1>124.5	76>70	53.2	61.7>50.4	62.7>70.4	57.8>49.3	50.7	75.1>66.4	42.1>45	35.6	26	21	23.7	37
Sodium [mmol/L]	140>135>135>134>139	140>141	140	138>139	135>138	137>139	136	144>143	152>150	139	140	134	139	141
Potassium [mmol/L]	3.7>6.1>6.1>4.5>3.4	3.5>3.8	-	3.3>3.6	3.1>3.3	3.3>3.7	3.5	4.1>3.3	3.6	4.3	3.9	3.8	4.4	4.2
Chloride [mmol/L]	106>107>109>110	112	112	108	104>106	100>101	99	107	114	103	100	97	101	103.6
Calcium [mmol/L]	2.25>1.95>2>1.56>1.66	1.78>1.77	1.82	1.87>1.81	1.83>1.8	1.88>1.94	1.77	1.89>1.86	1.94	-	2.03	2.27	2.5	2.34
Albumin [g/L]	20.3>20.6>11.6>19.9>19.7	22.5>20.3	19.3	20.2	19.9>23.6	24.6>24.7	24.8	24.9>25	25.3	23.8	33.3	42.1	46.6	30
PT [SEC]	35.2>32.6	24.6	22.8	24.8	-	21.7>16.7	30.5	21	13.5>17.9	16.7	16.9	15.5	10.3	10.3
PTT [SEC]	43.3>45.3	40.3	37.8	40.8>33.8	-	44.8>40.3	46.6	44.4	35	42.1	36.5	37.8	-	-
INR [%]	1.4>2.61>4.72>2.41	2.18>1.8	1.66	1.81>1.78	2	1.58>1.3	2.25	1.54>1.95>2.68>4.74	1>1.3	1.3	1.4	1.2	0.8	0.95

## Discussion

Burn’s related morbidity and mortality can be anticipated from the age of the patient and the size of the body being involved. [1,2,7] The older the patient with burn injury, the higher the morbidity and mortality due to physiological reduction of immune function and thinner skin [6]. Furthermore, the size of the burns is an independent factor for the burns’ related morbidity and mortality, which can be calculated and measured through the TBSA [1,2]. The larger the surface area involved, the greater the morbidity and mortality [9,10]. Jeschke et al estimated that TBSA of more than 40% is associated with a high risk of morbidity and mortality [6]. With a proper understanding of the burn’s physiology, a higher rate of survival can be achieved with even higher TBSA of burns. There are no previous studies in the literature of cases with a TBSA of this case and surviving thereafter in Saudi Arabia. We present a case of a 24-year-old Somali male patient who survived a 95% TBSA burns that is second degree. The approach we implemented for the patient for his survival is detailed next.

Fluid resuscitation

Immediate and accurate assessment of the depth as well as the size of burns and proper fluid management help in reducing patient morbidity and mortality significantly [7]. Fluid resuscitation is one of the most important primary goals for early treatment of thermally injured patients. It improves survival significantly and is generally required for patients with TBSA more than 20% [8,11]. The resuscitation usually starts with Ringer's lactate due to its close composition to the extracellular fluid. After 12-14 hours, colloids with either albumin or plasma will be administered. After 24 hours, 5% dextrose in water is administered to replace the massive evaporative water loss [11,12]. For our patient, upon his arrival, the TBSA was calculated and he was put on oxygen mask. Also, fluid resuscitation with 1L Ringer's lactate was started and then shifted to plasma 500 mL. Plasma is generally advised because of the lower requirements of fluid volume within 24 hours as well as because it lowers the risk of increased intra-compartmental pressure and the risk of developing abdominal compartment syndrome [1,2]. 24 hours later, he was on 300 mL/hour of 5% dextrose solution. Furthermore, IV route of administration of pethidine was done to relieve the pain, as other routes of administration have unpredictable uptake [1]. Furthermore, IV metoclopramide and hydrocortisone were administered initially. The patient was acidotic upon ER admission, with reduced partial pressure of oxygen. However, these parameters improved within 1 hour after resuscitation. Essentially, the urine output (UOP) has to reach 30-50 mL/hour post resuscitation [10]; our patient’s UOP reached 70 mL/hour after resuscitation.

Escharotomy

Eschar is an avascular necrotic tissue, which is considered a protein-rich environment for microbial colonization. Following burns, eschar might develop. It decreases the perfusion to the tissues, thus it needs to be decompressed to restore the circulation towards the limbs under a procedure called escharotomy [8]. Our patient underwent escharotomy immediately for all four limbs, followed by application of wound dressing.

Burn shock

Burn shock can result if TBSA reaches more than 20% due to the resultant hypovolemia and the systemic effects from the released cytokines and other inflammatory mediators, which can result in significant cardiovascular dysfunction [7,13]. Our patient had intravascular hypovolemia and shock, which was managed by warmed normal saline that was then changed to 300 ml/hour 5% dextrose solution. Furthermore, inotropes were titrated to keep the MAP more than or equal 65 mmHg.

Disseminated intravascular coagulation

Disseminated intravascular coagulation (DIC) can develop following severe burns, especially in TBSA that is more than 20%. This is due to the release of tissue factors from the damaged cells, which result in the activation of the extrinsic pathway in the coagulation system which results in intravascular coagulation and thrombin generation. Hyperfibrinolysis also occurs along with hypercoagulation, evident by the increase in prothrombin time (PT), activated partial thromboplastin time (aPTT), D-dimer, and low fibrinogen [14]. Our patient developed DIC on the third day after the admission, with elevated PT and aPTT levels on laboratory results. He was treated with four units of warmed FFP.

It was observed that hemoglobin and total bilirubin levels were elevated, which can be explained due to the resultant hypovolemia.

Literature review

Table 4 summarizes nine cases found in the literature about thermal burns with large TBSA, most of whom have survived thereafter. One patient was more than 55 years old, and all other patients including in this case are below 55 years of age. One patient was an infant of 4 months and one was a pregnant woman. The majority of the cases had a TBSA of 80% or more, with the majority having full-skin thickness degree burns. Although almost all patients were having systemic complications, the majority survived and returned to do their daily living activities.

**Table 4:** Summary of the cases describing patients injured by thermal burns, with TBSA and their prognosis. TBSA: total body surface area; MODS: Multi-Organ Dysfunction Syndrome; ARDS: Acute respiratory distress syndrome

Paper	Patient	Accident	TBSA	Prognosis
Hahn [15]	A 40-year-old man, plumber worker.	Injury by hot water and hot steam during replacement of hot water plumb.	80% TBSA with depth of superficial and deep-second degree burns.	Patient complicated with colitis, drug induced colorectal bleeding, and post-traumatic tinnitus. He survived, and returned to work within 92 weeks, with 50% ability to work.
Thomson et al. [16]	A 4-months-old female infant.	House fire with severe smoke inhalation injury.	85% TBSA with depth of 80% full skin thickness.	Patient complicated with sepsis, ARDS, pneumonia, and generalized osteoporosis. She survived, and returned home and adapted well after 6 months of concentrated rehabilitation.
Robenpour et al. [17]	A 21-year-old man.	Patient injured by gas explosion.	95% TBSA with the mixture depth of deep-second and full skin thickness.	Patient complicated with sepsis, burn shock, and as in severe catabolic state. He survived, and returned home within 18 months, and is capable to carry out all activities of daily living well.
Janis [8]	A 58-year-old female.	Patient injured during house explosion with resultant fire.	63% TBSA with depth of full skin thickness primarily.	Patient survived and returned home after 3 weeks of rehabilitation, where she lives independently and continues to improve. No data are available for the mentioned complications.
Liu et al. [18]	No data are provided.	Patient injured during flame burn with inhalational injury.	100% TBSA with depth of 96% full skin thickness.	Patient survived with successful healing of the burn. No data are provided for the complications.
Chai et al. [19]	Total of 8 patients, 4 males and 4 females, aging (22-45) years old.	Patients injured during an aluminum dust explosion accident.	55%-98% TBSA with depth of 45%-97% full skin thickness.	1 patient died, and the other 7 patients survived with successful healing of the burn.
Guo et al. [20]	Total of 30 patients, 21 males and 9 females, aging (17-48) years old.	Patients injured mostly by flame, followed by explosion, then scald.	88%-96% TBSA with depth of 62%-88% full skin thickness.	21 patients died mostly by sepsis, and the other 9 patients survived with successful healing of the burn. Survived patients complicated by sepsis, MODS, and burn shock.
Li et al. [21]	A 29-year-old male.	Patient was injured by molten steel that is nearly 1,500 degrees, in a furnace explosion, from the top of the head and through the whole body.	99.5% TBSA with depth being 5.5% of deep-second, 71% third degree, and 23% fourth degree.	Patient survived, but lost the ability to walk and hold objects due to contracted scars and amputation, with left eye vision impairment. However, with the support of his family he is willing to live and face the reality.
Caso et al. [22]	A 30-year-old female, pregnant in her 34^th^ week.	Patient was assaulted with an accelerant resulting in burn injuries.	75% TBSA with depth of deep-partial and full skin thickness.	Patient developed pneumatosis intestinalis and multiple operations were done, but she survived and discharged on day 61.

## Conclusions

Mortality and morbidity in burns are associated with the total body surface area (TBSA), especially for burns with TBSA of more than 40%. Patients with large burn areas need a proper estimation of the TBSA with early and careful fluid resuscitation, as it is one of the primary goals for the treating physician. This reduces morbidity and mortality significantly. Early and immediate surgical intervention is with escharotomy with the application of antimicrobial dressing. We present a case of a patient presenting to our hospital with 95% TBSA burn, who survived and improved and was discharged home and is now living comfortably. As shown from the approach that we have implemented and as presented from the literature review, we conclude that patients with burns of large TBSA must be treated in specialized centers for better outcome and survival.
